# A Study on a Neural Network Risk Simulation Model Construction for Avian Influenza A (H7N9) Outbreaks in Humans in China during 2013–2017

**DOI:** 10.3390/ijerph191710877

**Published:** 2022-08-31

**Authors:** Wen Dong, Peng Zhang, Quan-Li Xu, Zhong-Da Ren, Jie Wang

**Affiliations:** 1 Faculty of Geography, Yunnan Normal University, Kunming 650500, China; 2 GIS Technology Engineering Research Centre for West-China Resources and Environment of Educational Ministry, Yunnan Normal University, Kunming 650500, China; 3 College of Intelligent Information Engineering, Chongqing Aerospace Polytechnic College, Chongqing 400021, China; 4 State Key Laboratory of Estuarine and Coastal Research, East China Normal University, Shanghai 200241, China; 5 Chongqing City Management College, Chongqing 401331, China

**Keywords:** H7N9, GIS, spatial analysis, risk factors, risk simulation model

## Abstract

The main purposes of this study were to explore the spatial distribution characteristics of H7N9 human infections during 2013–2017, and to construct a neural network risk simulation model of H7N9 outbreaks in China and evaluate their effects. First, ArcGIS 10.6 was used for spatial autocorrelation analysis, and cluster patterns ofH7N9 outbreaks were analyzed in China during 2013–2017 to detect outbreaks’ hotspots. During the study period, the incidence of H7N9 outbreaks in China was high in the eastern and southeastern coastal areas of China, with a tendency to spread to the central region. Moran’s I values of global spatial autocorrelation of H7N9 outbreaks in China from 2013 to 2017 were 0.080128, 0.073792, 0.138015, 0.139221 and 0.050739, respectively (*p* < 0.05) indicating a statistically significant positive correlation of the epidemic. Then, SPSS 20.0 was used to analyze the correlation between H7N9 outbreaks in China and population, livestock production, the distance between the case and rivers, poultry farming, poultry market, vegetation index, etc. Statistically significant influencing factors screened out by correlation analysis were population of the city, average vegetation of the city, and the distance between the case and rivers (*p* < 0.05), which were included in the neural network risk simulation model of H7N9 outbreaks in China. The simulation accuracy of the neural network risk simulation model of H7N9 outbreaks in China from 2013 to 2017 were 85.71%, 91.25%, 91.54%, 90.49% and 92.74%, and the AUC were 0.903, 0.976, 0.967, 0.963 and 0.970, respectively, showing a good simulation effect of H7N9 epidemics in China. The innovation of this study lies in the epidemiological study of H7N9 outbreaks by using a variety of technical means, and the construction of a neural network risk simulation model of H7N9 outbreaks in China. This study could provide valuable references for the prevention and control of H7N9 outbreaks in China.

## 1. Introduction

The first avian influenza A(H7N9) human case was found in China in early 2013, followed by the discovery of the virus in local live poultry markets [1]. Since then, avian influenza A (H7N9) outbreaks in humans (abbreviated to H7N9 outbreaks in this study) became a very important issue during H7N9 epidemics for Chinese health [2]. The avian influenza A (H7N9) virus is a form of influenza A caused by the H7N9 bird flu virus [3]. The body temperatures of most H7N9 human cases are above 38.5 ℃, and symptoms such as cough and expectoration may develop into severe pneumonia and death within one week [4]. In 2017, a mutant strain was considered to be highly pathogenic to birds and could lead to multiple H7N9 outbreaks in China [2]. Since the first H7N9 human case emerged in early 2013, there have been six H7N9 outbreaks in 29 provinces and cities in China, infecting about 1600 people [5]. H7N9 outbreaks have posed great challenges to public health and social and economic stability in China, and the study on the spatial and temporal distribution and the outbreak risk simulation of H7N9 human infections has become a research hotspot in the public health field in recent years.

In 2017 Chen et al. believed that the high pathogenicity and the rapid development of the H7N9 virus might be caused by frequent gene recombination and easy mutation in the breeding process [6]. In order to address the severe challenges posed by H7N9 outbreaks, a large number of experiments and vaccine studies were carried out in 2015 and 2016. In the fifth H7N9 outbreaks in humans in China, the number of people infected with H7N9 virus was increasing continuously, which attracted extensive attention from the public and public health departments [7]. In order to control H7N9 outbreaks, many areas along the southeast coast of China (such as Shanghai), gradually closed all live poultry markets after 4 April 2013 [8]. At present, studies have found that outbreaks and transmission of H7N9 outbreaks are liable to be related to poultry trade, vegetation cover, population density, temperature, rainfall and humidity [9]. Since 2013, H7N9 outbreaks in the Yangtze River Delta region and eastern China have been reported repeatedly [10,11]. Studies showed that as one of the three major migratory bird routes through China, the risk of transmission of H7N9 outbreaks in eastern China, including the Yangtze River Delta and Pearl River Delta, was particularly high [12,13]. In addition, the spatial distribution of H7N9 human cases in China showed that the density of H7N9 human cases was the highest from the vicinity of the Yangtze River Delta to the south of the pearl River Delta, forming a large space-time aggregation area [14].

H7N9 outbreaks have posed a serious threat to public health and social stability in China. Therefore, it is quite necessary to study the spatial distribution and transmission characteristics of H7N9 outbreaks. With the continuous development of computer technology, machine learning has been widely used in medical and public health research. Previous studies confirmed that neural network, random forest, support vector machine and other models were better than traditional statistical models in disease assessment to some extent [15,16]. One study suggests that using epidemiologically and environmentally dependent transmission rates can potentially generate more practical simulation results [17]. The main purposes of this study were to carry out epidemiological study and explore spatial distribution characteristics of H7N9 outbreaks based on the spatial autocorrelation analysis during 2013–2017, and to construct a neural network risk simulation model of H7N9 outbreaks in China by considering environmental factors and evaluate their effects. The research results could provide valuable references for the prevention and control of N7H9 outbreaks in China.

## 2. Materials and Methods

### 2.1. Data Collection

Data of H7N9 human cases from 2013–2017 were obtained by applying to the Chinese CDC (Centers for Disease Control) Information Center. The poultry data, breeding data and livestock production value data were from the Chinese National Bureau of Statistics (http://www.stats.gov.cn, accessed on 11 August 2020), and the market data and output value data of poultry in different provinces of China Rural Statistical Yearbook from 2013–2017 were downloaded online. Stream vector data were obtained from the Institute of Geographic Sciences and Natural Resources Research of the Chinese Academy of Sciences (http://www.resdc.cn/data.aspx?DATAID=221, accessed on 11 August 2020). The vegetation index data came from the National Data Center for Tibetan Plateau Science (http://data.tpdc.ac.cn, accessed on 11 August 2020).

### 2.2. Research Methods

#### 2.2.1. Spatial Autocorrelation Analysis

Spatial autocorrelation analysis includes global spatial autocorrelation and local spatial autocorrelation. Global spatial autocorrelation and local spatial autocorrelation reflect the correlation of research objects at global and local scales respectively. Global spatial autocorrelation analysis can be used to determine whether cases are clustered or discrete at the domestic observation scale [18]. Local spatial autocorrelation analysis can reflect the spatial variation characteristics of cases. Four types of clusters can be obtained through local spatial autocorrelation, the distribution of these four types of clusters respectively reflects the H-H cluster (H-H, high value surrounded by high value), the H-L cluster (H-L, high value surrounded by low value), the L-H cluster (L-H, low value surrounded by high value) and the L-L cluster (L-L, low value surrounded by low value) [19]. In this study, ArcGIS 10.6 was used for spatial autocorrelation analysis, and H-H cluster patterns of H7N9 outbreaks were analyzed in China during 2013–2017, in order to explore hotspots of outbreaks.

#### 2.2.2. Neural Network Risk Simulation Model of H7N9 Outbreaks

##### Sample Collection

**Figure 1:**
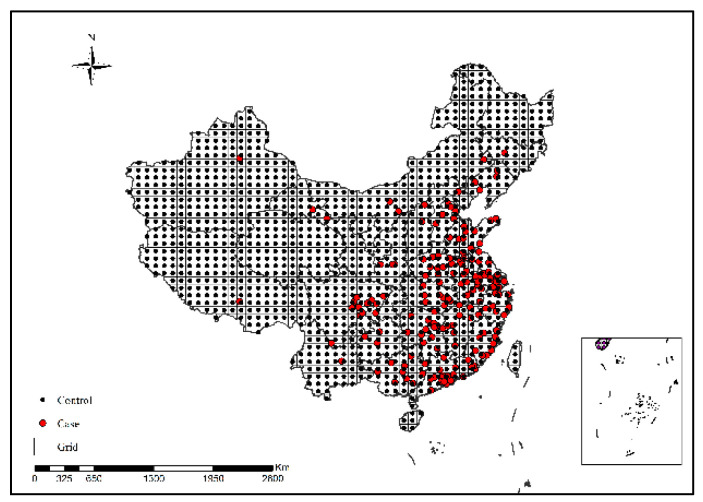
Sample collection results.

As shown in Figure 1, sample collection rules adopted in this study were as follows: (1) a total of 1474 H7N9 human cases in China were collected from 2013 to 2017, (including 155 cases in 2013, 326 cases in 2014, 196 cases in 2015, 243 cases in 2016, and 554 cases in 2017); ②a map of China (1: 4,000,000) was regularly divided into 100 km × 100 km grids, and the center of gravity of the grids was extracted. Each grid center of gravity that did not coincide with any case point was used as a control sample [20], and a total of 223 control sample points were obtained.

##### Neural Network Modeling

First, SPSS 20.0 was utilized to analyze the correlation of population, livestock production value, poultry farming, poultry market, vegetation index, distance between case sites and rivers and other influencing factors of H7N9 outbreaks in China. The quantitative data were described by mean ± standard deviation (X ± S). Qualitative data were described in relative numbers, and the χ2 test was used for comparison between groups, and *p* < 0.05 was considered statistically significant.

In this study, the collected sample points were divided into training set and test set by 7:3, and the risk factors related to the epidemic in the correlation analysis were incorporated into the neural network model, then a neural network risk simulation model of H7N9 outbreaks in China was constructed iteratively through neural network fitting. The neural network adopted in this study had four layers, with the number of neurons in each layer being 100, 50, 10 and 1 respectively. The activation functions of the first three layers are all rectified linear units (ReLUs). For fully connected neural networks, the ReLU function can ensure the global convergence of gradient descent [21], thus avoiding the problems of gradient explosion and gradient disappearance. The ReLU formula is the following [22]:

$$f\left(x\right)=\left\{\begin{array}{c} 0,\;x\leq 0\\ x,\;x>0 \end{array}\right.$$

where *x* is the output vector of the neural network of the upper layer into the neuron. Since the purpose of this study was a binary classification, only one neuron was required for the last layer, so the sigmoid function was selected as the activation function. This function can map a real number in the interval (0,1), which is very suitable for dichotomy problems [23]. The sigmoid formula is the following [24]:

$$S\left(x\right)=\frac{1}{1+e^{x}}$$

where, *x* is the output vector of the neural network of the upper layer into the neuron, and e is the natural logarithm.

Since the learning rate might be controlled by the output error, the binary cross entropy loss function was selected as the loss function of the neural network model in this study, so that the sigmoid function could avoid the problem of the decrease in the learning rate of the mean square error loss function during the gradient descent [25]. The formula for the binary cross entropy loss function is the following [26]:

$$J\left(\theta \right)=-\overset{m}{\underset{i=1}{\sum }}y_{\left(i\right)}\log h_{\theta }\left(x_{\left(i\right)}\right)+\left(1-y_{\left(i\right)}\right)\log\left(1-h_{\theta }\left(x_{\left(i\right)}\right)\right)$$

where *x* represents the output vector of the neuron’s calculation results, *y* represents the label of the sample, 0 or 1, and h represents the activation function. The learning rate in this study was set to 0.01, and the number of iterations was 50. TensorFlow 2.1 was used to construct a neural network risk simulation model of H7N9 outbreaks in China in this paper.

## 3. Results

### 3.1. Spatial Pattern of H7N9 Outbreaks in China

**Figure 2:**
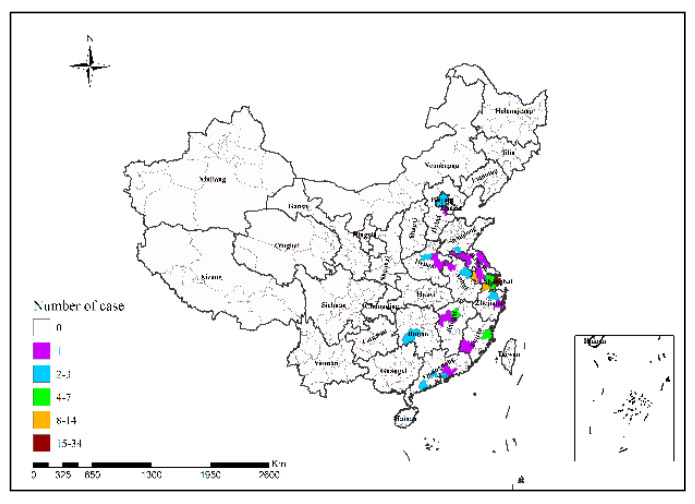
Spatial pattern of H7N9 outbreaks in China in 2013.

**Figure 3:**
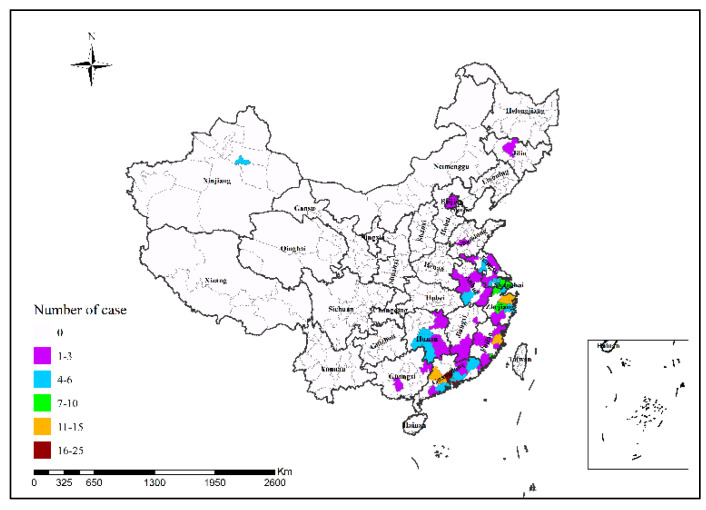
Spatial pattern of H7N9 outbreaks in China in 2014.

**Figure 4:**
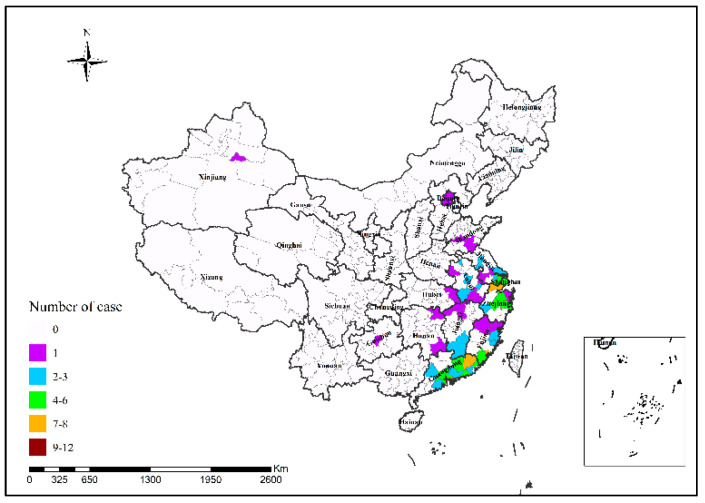
Spatial pattern of H7N9 outbreaks in China in 2015.

**Figure 5:**
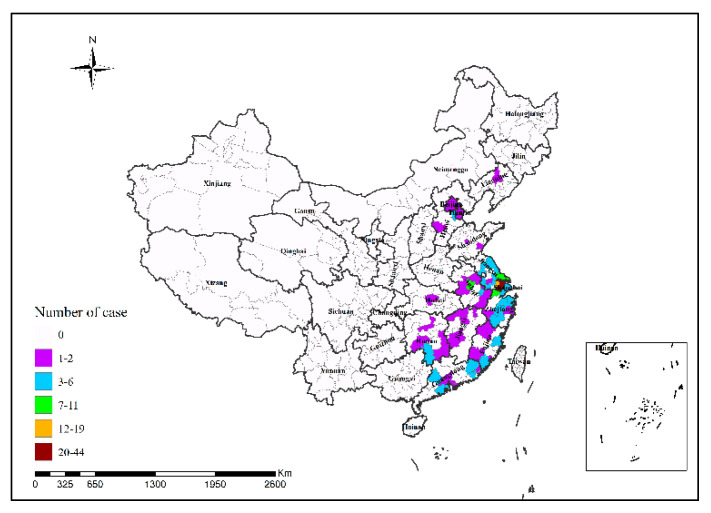
Spatial pattern of H7N9 outbreaks in China in 2016.

**Figure 6:**
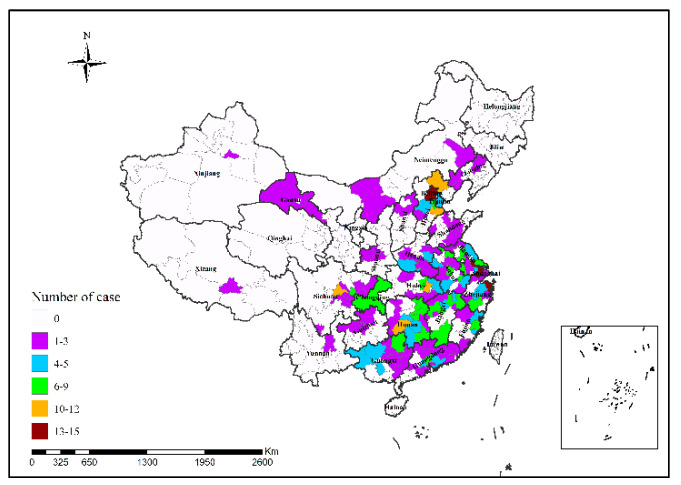
Spatial pattern of H7N9 outbreaks in China in 2017.

In 2013, Shanghai had the highest number of H7N9 human infections (34 cases); in 2014, Guangzhou had the highest number of H7N9 human infections (25 cases), followed by Shenzhen (24 cases); in 2015, Shenzhen had the highest number of H7N9 human infections (12 cases); in 2016, Suzhou had the highest number of H7N9 human infections (48 cases); in 2017, Ningbo and Taizhou had the highest number of H7N9 human infections (15 cases in both cities), followed by Suzhou (14 cases) and Beijing (13 cases), as shown in Figure 2, Figure 3, Figure 4, Figure 5 and Figure 6. During the study period, the incidence of H7N9 outbreaks in China was high in the eastern and southeastern coastal areas of China, with a tendency to spread to the central region.

### 3.2. Spatial Autocorrelation Analysis

#### 3.2.1. Global Spatial Autocorrelation Analysis of H7N9 Outbreaks

Global Moran’s I statistics were used to evaluate whether the cumulative number of confirmed H7N9 human cases in each region is spatially relevant. Moran’s I statistics test significance based on a Monte Carlo simulation of a stochastic permutation process, and I ranges from −1 (dissimilar value clustering) to +1 (similar value clustering), with 0 indicating that there is no spatial autocorrelation [27]. Table 1 shows results of global spatial autocorrelation analysis and significance test of H7N9 outbreaks in China from 2013 to 2017. Moran’s I values of global spatial autocorrelation of H7N9 outbreaks in China from 2013 to 2017 were 0.080128, 0.073792, 0.138015, 0.139221 and 0.050739, respectively, which were statistically significant (*p* < 0.05). Therefore, the spatial distribution of cases during the 5-year study period was not random, but there was obvious spatial autocorrelation during the epidemics in these five years. In the global spatial autocorrelation analysis, Moran’s I values of H7N9 outbreaks were all greater than 0, indicating that the epidemic was positively correlated in space, and the epidemic occurrence had a spatial clustering trend.

#### 3.2.2. Local Spatial Autocorrelation Analysis of H7N9 Outbreaks

**Figure 7:**
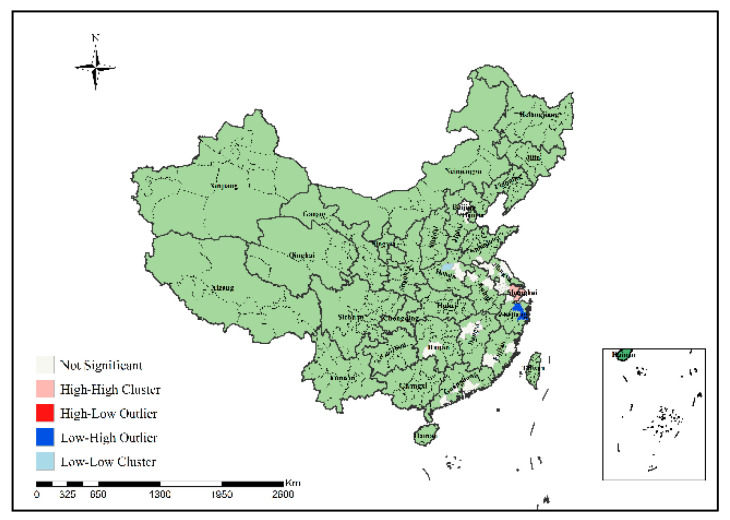
Local spatial autocorrelation analysis results of H7N9 outbreaks in China in 2013.

**Figure 8:**
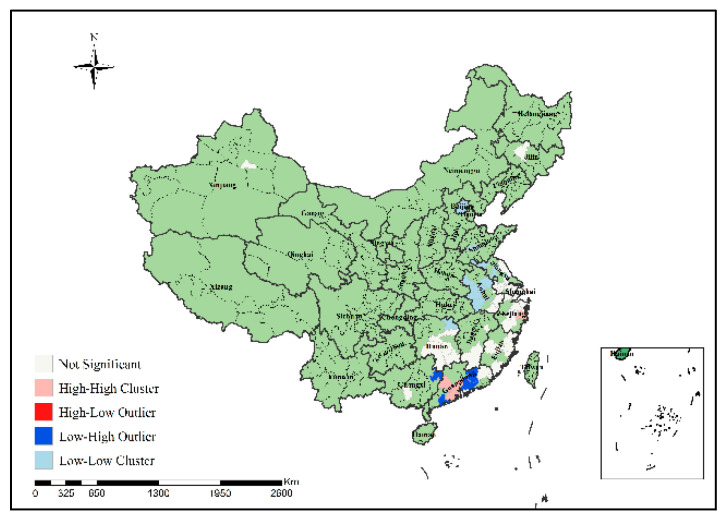
Local spatial autocorrelation analysis results of H7N9 outbreaks in China in 2014.

**Figure 9:**
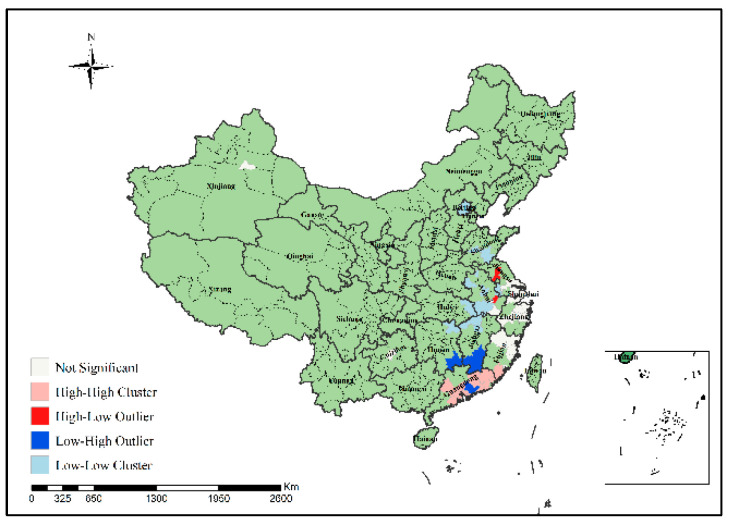
Local spatial autocorrelation analysis results of H7N9 outbreaks in China in 2015.

**Figure 10:**
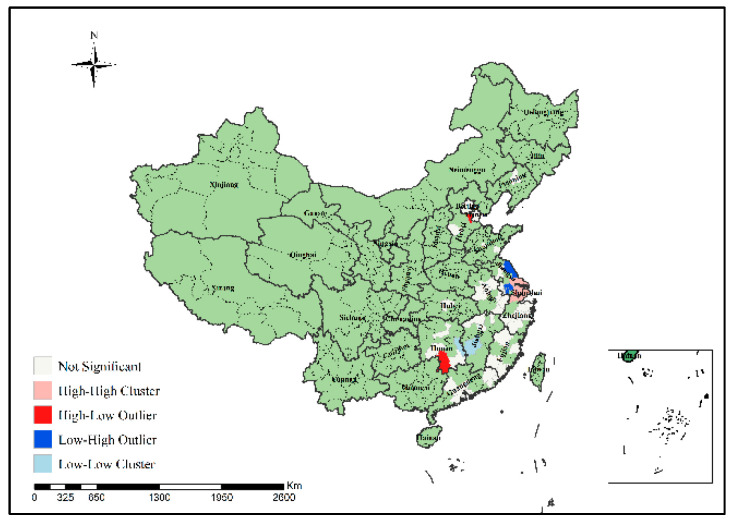
Local spatial autocorrelation analysis results of H7N9 outbreaks in China in 2016.

**Figure 11:**
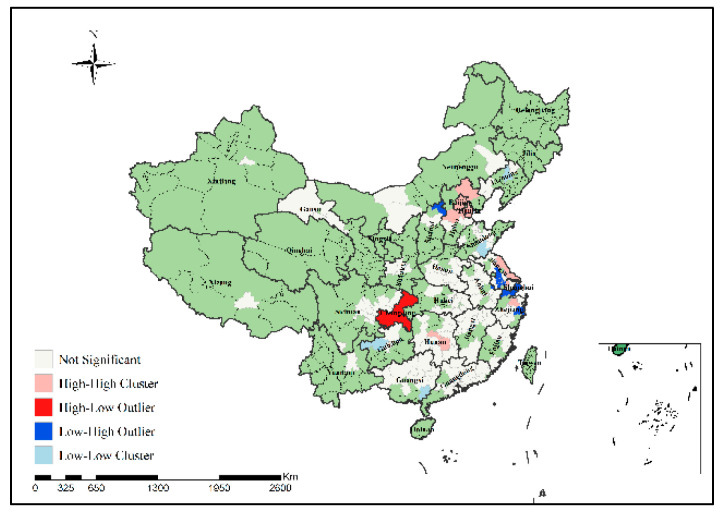
Local spatial autocorrelation analysis results of H7N9 outbreaks in China in 2017.

In order to specifically identify where the H7N9 epidemic clusters and outliers occurred, this study further conducted local spatial autocorrelation analysis of H7N9 outbreaks in China from 2013 to 2017 to obtain the visual clustering distribution map of the epidemic, as shown in Figure 7, Figure 8, Figure 9, Figure 10 and Figure 11. As can be seen from figures, local spatial autocorrelation analysis of H7N9 outbreaks further confirmed the high-risk areas of the study area, and found that the hot spots with high incidence were relatively concentrated. There were four kinds of outbreak clusters or outliers during the study period: the high-high (H-H) cluster, the high-low (H-L) outlier, the low-high (L-H) outlier, and the low-low (L-L) cluster. The H-H cluster was a high value cluster pattern of outbreaks that needed to be paid attention to, indicating that both H-H and its surrounding areas had high incidence numbers. Local spatial autocorrelation analysis of H7N9 outbreaks in China from 2013 to 2017 showed that: the locations of high value clusters were relatively concentrated, and the H-H cluster region was the high-risk area of the epidemic, mainly distributed in Jiangsu, Hunan, Zhejiang, Guangdong, Beijing and Shanghai during the study period. Specifically, in 2013 the H-H cluster areas were in Shanghai, Jiaxing, Suzhou and Wuxi; in 2014, the H-H cluster areas were in Taizhou, Zhaoqing, Foshan, Guangzhou, Jiangmen and Zhongshan; in 2015, the H-H cluster areas included Zhangzhou, Chaozhou, Meizhou, Heyuan, Haifeng, Jieyang, Zhaoqing, Foshan, Guangzhou, Dongguan, Shenzhen, Jiangmen and Zhongshan; in 2016, the H-H cluster areas included Nantong, Taizhou, Wuxi, Suzhou, Huzhou, Jiaxing and Shanghai; in 2017, theH-H cluster areas included Chengde, Beijing, Baoding, Langfang, Tianjin, Yancheng, Nantong, Wuxi, Shaoxing, Loudi and Hengyang, as shown in Figure 7, Figure 8, Figure 9, Figure 10 and Figure 11. These H-H cluster areas with high concentrations of H7N9 outbreaks during the study period were characterized by spatial hot spots, which should be focused on in future epidemic prevention and monitoring.

In addition, the H-L outlier areas mainly occurred in part of Jiangsu, Anhui, Hunan, Chongqing and other places during the study period, indicating that the number of cases in the relevant areas was high, but the number of cases in the surrounding areas was low. The L-H outlier areas mainly occurred in part of Zhejiang, Guangxi, Hunan, Jiangxi, Shanxi, Jiangsu and Guangdong, indicating that the cases number in the relevant regions was low, but the cases number in the surrounding areas was high. The L-L cluster areas was the low value clustering pattern of the epidemic, which mainly occurred in part of Liaoning, Shandong, Guizhou, Guangxi, Beijing, Jiangsu, Anhui, Hunan, Shandong, Hubei and Jiangxi, showing that the related areas and their surrounding areas had the characteristics of low cases number. Although these H-L, L-H and L-L regions did not have a high concentration of outbreaks, they still need to be paid attention to in the epidemic prevention and control work.

#### 3.2.3. Neural Network Risk Simulation Model of H7N9 Outbreaks in China

In the data collection stage, we collected eight additional influencing factors, which were: urban population, provincial animal husbandry output value, provincial poultry breeding, provincial poultry market, NDVI, and distance between case points and rivers. In addition, we also considered the impact of the city on cases. Therefore, a total of nine risk factors were included in this study.

The reason for selecting the above factors was that the source of H7N9 outbreaks was still unclear, and it was presumed that the infection is mainly avian. The high-risk population was mainly workers engaged in avian-related work, so relevant data and information on agriculture and animal husbandry were needed. At the same time, wild birds in cities might also be carriers of the virus, and wild birds mainly lived in areas covered with vegetation, so the NDVI was taken into consideration. The main route of transmission of H7N9 outbreaks was avian secretions and excreta, so it could not be ruled out that this material might enter rivers and cause human infection.

Cities and urban populations were considered because it had been demonstrated that H7N9 outbreaks were the result of genetic recombination between wild birds from Southeast Asia and chickens from Shanghai, Zhejiang, and Jiangsu provinces in China. In addition, cattle and sheep were mainly eaten in northwestern and North China, which might also be risk factors for the diagnosis of H7N9 outbreaks. After confirming the above nine factors, we used the independent sample *t*-test in SPSS to determine whether these nine factors were correlated with confirmed cases (*p* < 0.05), and finally found that only three factors had a significant correlation with the diagnosis: urban population, urban average vegetation, and the distance from the case to the river.

For the construction of the neural network, after confirming the above three factors, we took the above three factors as independent variables and the diagnosis as the dependent variable to form a matrix of N*4 (N rows and four columns) and input it into the neural network. The goal of our model is to judge whether case data are confirmed or not, so our output is binary classification by a sigmoid function.

Given the above, an independent sample *t*-test was used to conduct a correlation analysis on possible epidemic influencing factors in this study, as shown in Table 2. The statistically significant epidemic influencing factors screened out by correlation analysis were population of the city, average vegetation of the city, and distance between case sites and rivers (*p* < 0.05), finally, these influencing factors were included in the neural network risk simulation model of H7N9 outbreaks in China.

**Figure 12:**
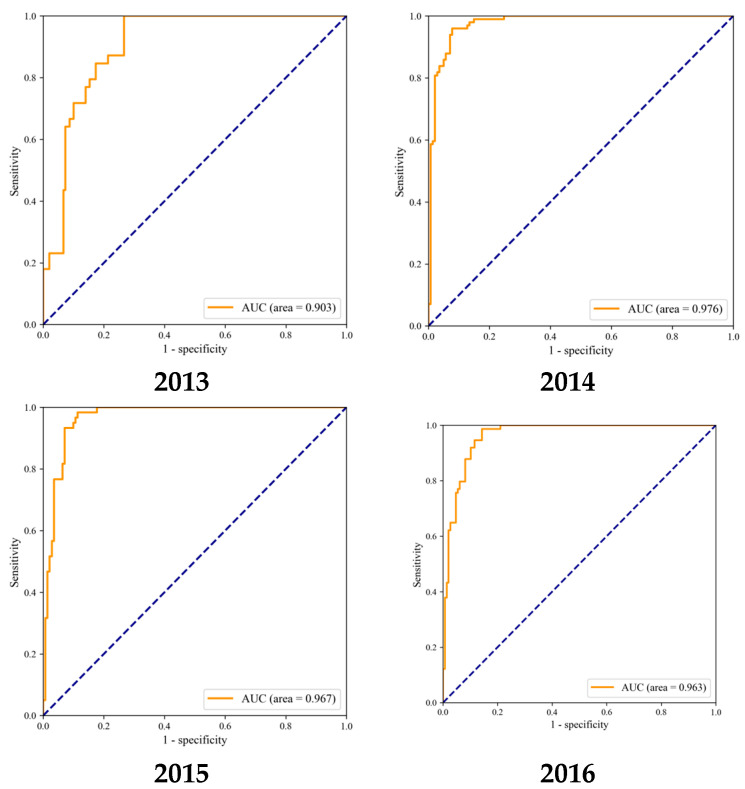

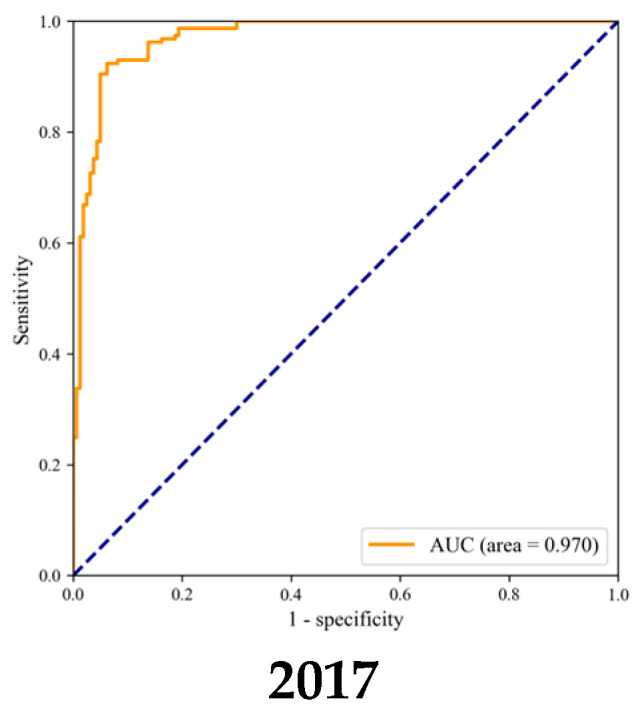
ROC curves of the neural network risk simulation model of H7N9 outbreaks in China from 2013 to 2017.

In this study, the assessment of consistency rate and AUC (area under the ROC curve, as shown in Figure 12) were used to evaluate indicators of the neural network risk simulation model of H7N9 outbreaks in China. This risk simulation model was established based on the neural network algorithm in the epidemic training set samples, and the risk simulation was conducted on the epidemic test set from 2013 to 2017. The simulation accuracy of the neural network risk simulation model of H7N9 outbreaks in China from 2013 to 2017 were 85.71%, 91.25%, 91.54%, 90.49% and 92.74, respectively, and the AUC were 0.903, 0.976, 0.967, 0.963 and 0.970, respectively, the results show that the model constructed in this paper has relatively good accuracy, as shown in Table 3.

In this study, sample data sets of H7N9 outbreaks in China from 2013 to 2017 were randomly divided into a training set and a test set (7:3). The training set was used to establish the neural network risk simulation model of H7N9 outbreaks in China, and the test set was used to test the risk simulation effect of the model. The probability simulation value of H7N9 outbreaks was calculated by the model, and the outbreak risk map of the test set was obtained based on the probability simulation value and inverse distance weight spatial interpolation method.

**Figure 13:**
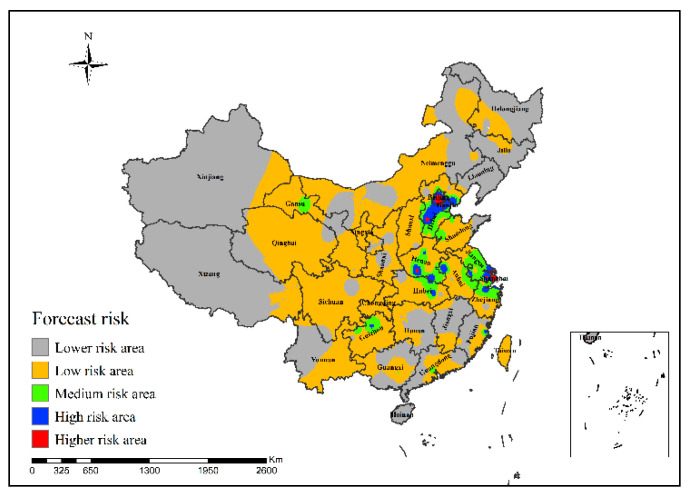
Risk simulation results of H7N9 outbreaks in China in 2013.

It can be seen from Figure 13 that in the epidemic risk map generated by the neural network risk simulation model of H7N9 outbreaks in China in 2013, the simulated areas with high risk of H7N9 outbreaks are mainly in Shanghai, Beijing and Hebei Province.

**Figure 14:**
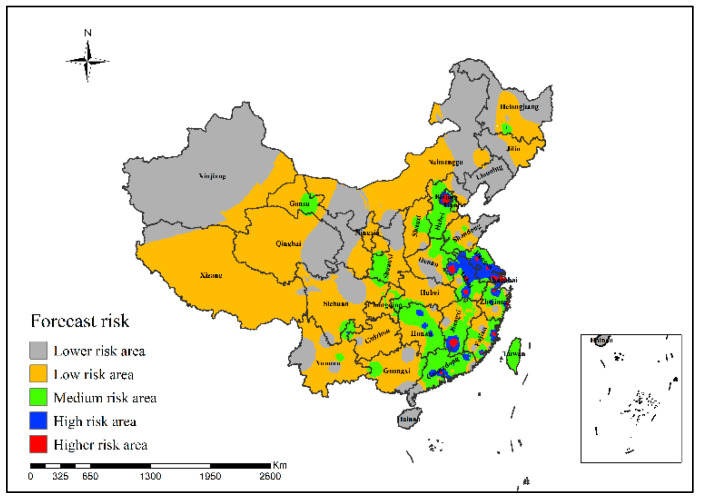
Risk simulation results of H7N9 outbreaks in China in 2014.

It can be concluded from Figure 14 that in the epidemic risk map generated by the neural network risk simulation model of H7N9 outbreaks in China in 2014, the simulated areas with high risk of H7N9 outbreaks were mainly concentrated in Beijing, Jiangsu, Zhejiang, Fujian and Guangdong provinces.

**Figure 15:**
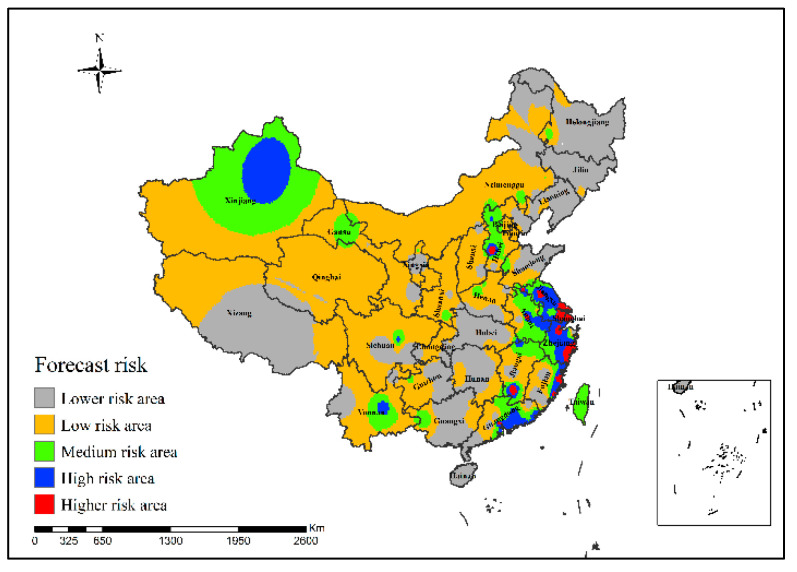
Risk simulation results of H7N9 outbreaks in China in 2015.

Figure 15 shows that in the epidemic risk map generated by the neural network risk simulation model of H7N9 outbreaks in China in 2015, the simulated high-risk areas of H7N9 outbreaks were mainly concentrated in Beijing, Jiangsu, Zhejiang, Fujian and Guangdong provinces of China, and the higher-risk areas did not change much since 2014.

**Figure 16:**
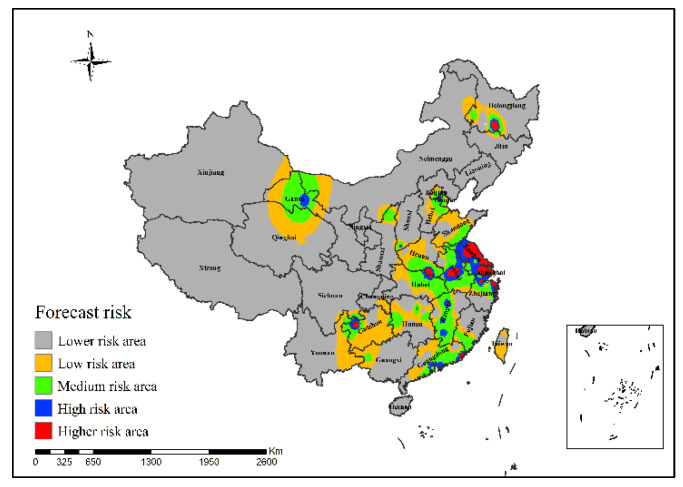
Risk simulation results of H7N9 outbreaks in China in 2016.

It can be concluded from Figure 16 that in the epidemic risk map generated by the neural network risk simulation model of H7N9 outbreaks in China in 2016, the high-risk areas of H7N9 human infections were mainly concentrated in Heilongjiang, Jiangsu, Zhejiang, Hubei, Henan, Jiangxi, Guizhou and Guangdong provinces, and the risk in Anhui, Shanghai and Jiangsu was more serious.

**Figure 17:**
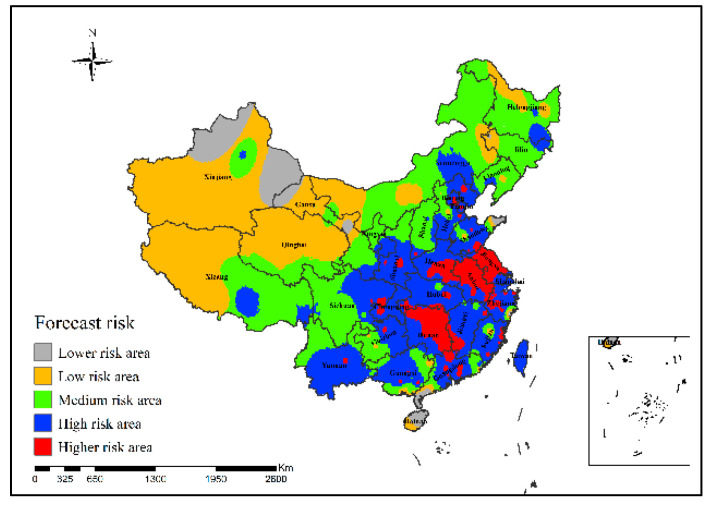
Risk simulation results of H7N9 outbreaks in China in 2017.

Figure 17 shows that in the epidemic risk map generated by the neural network risk simulation model of H7N9 outbreaks in China in 2016, the high-risk areas of H7N9 human infections were mainly concentrated in Heilongjiang, Jiangsu, Zhejiang, Hubei, Jiangxi, Guizhou and Guangdong provinces; among them, the risk in Hubei, Anhui, Shanghai and Jiangsu was more serious.

## 4. Discussion

In this paper, the epidemiological study of H7N9 outbreaks in China was conducted based on multiple methods. Firstly, this study used the complete case data of H7N9 outbreaks in China from 2013 to 2017 to analyze the spatial distribution and clustering of H7N9 outbreaks based on ArcGIS 10.6 software. Secondly, this paper used correlation analysis and a neural network model to simulate the risk of H7N9 outbreaks in China during the study period, so as to obtain the high-risk areas of the epidemic.

This study revealed that H7N9 outbreaks appeared in a total of 34 cities in China in 2013, among which Shanghai was the initial area of the epidemic and had the largest number of cases. By the end of 2017, a total of 143 cities had H7N9 outbreaks, among which Ningbo, Taizhou and Suzhou had the highest number of human cases. Since 2013, the epidemic had gradually spread spatially, with most cases occurring in 2017 and showing a trend of accelerating spread. In the global spatial autocorrelation analysis, Moran’s I values of H7N9 outbreaks were all greater than 0, and *p* values were all less than 0.05, indicating that the distribution of H7N9 outbreaks in China had a positive correlation. According to the cluster outlier analysis, the number of H-H regions of H7N9 outbreaks increased from four cities at the beginning to eleven cities in 2017, showing an obvious tendency of growth. It is suggested that high-risk areas of H7N9 outbreaks in China gradually increased during the study period and showed a trend of spreading from southeast to western and northern regions.

In view of the neural network model’s ability to accurately evaluate in data-based simulation and evaluation, this study used the model to simulate H7N9 outbreaks in China. Studies have shown that the evaluation ability of the neural network model is superior to random forest and logistic regression models and other modeling methods [28]. Some researchers have compared and analyzed artificial neural network with random forest and other classification models. For example, in terms of epidemic research, Wang et al. [29], Guo et al. [30], and Oliveira et al. [31] compared the efficiency of neural network, random forest and support vector machine models in the auxiliary diagnosis of AIDS and COVID-19, and results showed that the accuracy of the neural network model is higher than that of other models. In studies on other diseases, Yu et al. [32], Choi et al. [33], Lai et al. [34], Shh et al. [35] all made a comprehensive comparison among neural network, random forest and support vector machine in their respective studies, and concluded that the neural network model was more accurate than random forest and support vector machine models. Based on these research results and considering that the original data of this study were continuous data, the neural network model which might achieve better simulation effects was finally selected for modeling and simulation in this paper. Therefore, variables such as population, output value of animal husbandry, poultry breeding, poultry market, vegetation index, distance between case sites and river, and city were integrated, and statistically significant factors (city population, city average vegetation, and distance between case sites and river) in epidemic correlation analysis were incorporated into the neural network risk simulation model of H7N9 outbreaks in China (*p* < 0.05) in this study. The neural network risk simulation model of H7N9 outbreaks in China was constructed to simulate the risk areas of H7N9 outbreaks in China; the simulation accuracy was 73.12%, and the AUC was 0.812. The results show that the model established in this study has a good simulation effect on the risk of H7N9 outbreaks in China.

The neural network risk simulation model of H7N9 outbreaks in China established in this paper showed that the eastern and southeastern coastal areas of China were high-risk areas for H7N9 outbreaks, and the trend of spreading to southwest and north China was also shown. Shanghai and parts of Guangdong were always high-risk areas. Shanghai was the city with the first H7N9 outbreak, and although the cases had decreased during the study period, the risk continues to be high [36]. Parts of eastern China, including Anhui, Jiangxi, Henan, Shandong and Hubei, also contained relatively few localized high-risk areas. The outbreak of H7N9 outbreaks in southeastern coastal areas (Shanghai, Guangzhou, Shenzhen, etc.) might be due to the local climatic conditions suitable for the survival of H7N9 virus. In addition, there are many live poultry processing factories and farms in some areas along the southeast coast, and their live poultry breeding system generally adopts a semi-mixed breeding method. As poultry was the traditional carrier of the H7N9 virus, this live poultry breeding system may offer an ideal environment for the spread of the epidemic. All this increases the likelihood of local residents infected by the H7N9 virus through contact with poultry [37]. The region around the Yangtze River Delta also shows a high risk of infection. Most of the above regions were home to migratory birds and had high vegetation coverage, which was very suitable for wild birds, providing a good natural environment for the spreading of H7N9 virus.

## 5. Conclusions

The strengths of this paper were that the spatial distribution characteristics and the spread regularity of H7N9 outbreaks were studied from 2013 to 2017 in China based on GIS spatial analysis, correlation analysis and a neural network model, and finally a neural network risk simulation model of H7N9 outbreaks in China was established to simulate the outbreak of high-risk areas. Because the spread of H7N9 outbreaks was quite a complex process, some factors were difficult to be included and quantified in the model (such as government policy), and this might influence the model’s accuracy to a certain extent. Further studies are also required to delineate the mechanisms of H7N9 outbreaks’ transmission. Above all, the results show that the model established in this study achieved a good simulation effect of H7N9 outbreaks in China, which could provide a valuable reference and auxiliary decision support for the prevention and control of H7N9 human infections in China.

## Figures and Tables

**Table 1:** Results of global spatial autocorrelation analysis and significance test of H7N9 outbreaks in China from 2013 to 2017.

Year	Moran’I	*p*
2013	0.080128	0.047867
2014	0.073792	0.000089
2015	0.138015	<0.01
2016	0.139221	<0.01
2017	0.050739	0.042006

**Table 2:** Correlation analysis results.

Independent Variable	*p*	t	Mean Value ± Standard Deviation	95%CI	
				Lower	Upper
population	<0.001	−5.535	−0.70 ± 0.012	−0.094	−0.045
animal husbandry output value	0.359	1.780	0.041 ± 0.023	−0.004	0.086
poultry farming	0.871	−0.513	−0.011 ± 0.022	−0.055	0.032
poultry market	0.184	−4.685	−0.704 ± 0.015	−0.105	−0.043
mean vegetation	0.009	1.691	0.028 ± 0.016	−0.004	0.061
distance between case and river	<0.001	4.376	0.047 ± 0.010	0.026	0.068
city	0.181	−2.534	−0.062 ± 0.024	−0.111	−0.141

**Table 3:** Simulation results of the neural network risk simulation model of H7N9 outbreaks in China.

	2013	2014	2015	2016	2017
simulation accuracy	85.71%	91.25%	91.54%	90.49%	92.74%
AUC	0.903	0.976	0.967	0.963	0.970

## Data Availability

Data of avian influenza A(H7N9) human cases from 2013-2017 were obtained by applying to the Chinese CDC (Centers for Disease Control) Information Center. The poultry data, breeding data and livestock production value data were from the Chinese National Bureau of Statistics (http://www.stats.gov.cn), and the market data and output value data of poultry in different provinces of China Rural Statistical Yearbook from 2013-2017 were downloaded online. Stream vector data were obtained from the Institute of Geographic Sciences and Natural Resources Research of Chinese Academy of Sciences (http://www.resdc.cn/data.aspx?DATAID=221). The vegetation index data came from the National Data Center for Tibetan Plateau Science (http://data.tpdc.ac.cn).
